# Process evaluation of TXT2BFiT: a multi-component mHealth randomised controlled trial to prevent weight gain in young adults

**DOI:** 10.1186/s12966-016-0329-2

**Published:** 2016-01-19

**Authors:** Stephanie R. Partridge, Margaret Allman-Farinelli, Kevin McGeechan, Kate Balestracci, Annette T.Y. Wong, Lana Hebden, Mark F. Harris, Adrian Bauman, Philayrath Phongsavan

**Affiliations:** School of Life and Environmental Sciences, Charles Perkins Centre, The University of Sydney, Sydney, NSW 2006 Australia; Sydney School of Public Health, Charles Perkins Centre, University of Sydney, Sydney, NSW 2006 Australia; Centre for Primary Health Care and Equity, University of New South Wales, Sydney, 2052 Australia

**Keywords:** Young adults, Obesity prevention, Nutrition, Lifestyle, mHealth, Process evaluation

## Abstract

**Background:**

TXT2BFiT was one of the first few innovative mHealth programs designed for young adults (18–35 years) with demonstrated efficacy in weight management. However, research is lacking to understand intervention effectiveness, especially in complex, multi-component mHealth programs. This paper investigates participant perceptions of and engagement with the mHealth program components in the TXT2BFiT to understand program effects.

**Methods:**

Process evaluation data were collected continuously for the study duration. The TXT2BFiT program was a multi-component lifestyle program delivered intensively for 3-month followed by a 6-month maintenance phase. Program components included personalised coaching calls, text messages, emails, smartphone apps and website access. Process evaluation measures included frequency of use of components and frequency for number of components used (online survey data); dose delivered and engagement with program components (researcher logs and web platform reports); frequency, timing and difficulties experienced with program components (online survey data) and overall perceptions of program components (online survey data and semi-structured telephone interviews). Qualitative data analysis was performed using NVivo10.

**Results:**

Over 80 % of participants completed post-intervention (3-months, intervention, *n* = 110, control *n* = 104) and follow-up surveys (9-months, intervention, *n* = 96, control *n* = 104). Thirty intervention participants completed semi-structured telephone interviews. Participants reported high use of coaching calls, text messages and emails and no issues in content delivery from these components. These components were described as helping them to achieve their goals. Website and app use and engagement was low for the duration of the program. Participants would prefer incorporation of the self-monitoring apps and website resources into one smartphone application that can be individualised by entry of their personal data.

**Conclusions:**

Our process evaluation has allowed a comprehensive understanding of use and preference for different program components. The high value placed on the coaching calls is consistent with a desire for personalisation of the mHealth program and even further tailoring of text messages and emails. The findings of this study will be used to revise TXT2BFiT for future users.

**Trial registration:**

The trial is registered with the Australian New Zealand Clinical Trials Registry (ACTRN12612000924853).

## Background

Young adults aged 18–35 years are highly susceptible to weight gain [1–3], with the highest incidence of overweight and obesity compared to other age groups [4]. This is believed to be driven by their interactions with the obesogenic environment leading to unhealthy lifestyle behaviours such as poor dietary behaviours [5, 6] and sedentary lifestyles [7, 8], often associated with influence of digital technology [9].

While there may be negative impacts from the explosion of technology, this also provides opportunities for innovative and real-time delivery of health-related information. Smartphone ownership has increased substantially in recent years, with 65 % of Australians [10] and 64 % of Americans [11] owning a smartphone. Young adults record highest ownership at 85 % in the USA [12]. About three-quarters of 18–29 year old smartphone owners have used their phone in the last year to seek information about a health condition [12]. Smartphone intervention delivery, or mobile-health (mHealth) [13], could potentially provide a cost-effective intervention channel that allows real-time delivery of health information and serves as an effective tool for behaviour change to a wide ranging population [14].

mHealth or electronic-health (eHealth) programs have demonstrated success in the promotion of a range of healthy behaviours, including weight management [14, 15]. However, our recent systematic review showed only six of 22 interventions for weight gain prevention in young adults used new technology [16]. Of these six, only three studies reported the reasons why technology achieved or failed to achieve the desired outcomes of the interventions [17–19].

Process evaluation is an important step for understanding intervention effectiveness, especially in complex, multi-component mHealth programs [20]. Identifying and understanding how participants engage with and use the different components with respect to both the form of technology e.g. social media platforms, text messaging and smartphone apps and the content delivered can inform program improvement and ultimately, its effectiveness. Process evaluation can give insights to possible underlying factors for why a program fails or succeeds in effecting change [21]. Assuring that there is maximum participant engagement and satisfaction with program components can also enhance program retention [22].

Two recent process evaluations of technology-focused interventions in young adults have been published, beginning an evidence base to inform most effective program delivery to young adults [23, 24]. Both studies quantitatively reported how engagement with social networking intervention components was highly variable and declined over time. However, qualitative insights from Merchant et al., found many participants passively engage with social networking, not captured in quantitative engagement data [24].

TXT2BFiT was a multi-component mHealth lifestyle program custom designed for young adults (18–35 years) [25]. The program was delivered intensively for 3-months with personalised coaching calls, text messages, emails, smartphone apps and website access, followed by a 6-month maintenance phase. Program efficacy was tested in a randomised controlled trial (RCT) design, with weight reduction and improvements in lifestyle behaviours seen at 3-months [26] and maintained at 9-months (data unpublished). TXT2BFiT was one of the first few innovative mHealth programs. We also conducted a process evaluation of TXT2BFiT to gain understanding of program engagement, program fidelity and program component contribution to observed effects. Specifically, this paper investigates participant perceptions of and engagement with the mHealth program components in the TXT2BFiT to understand program effects.

## Methods

### TXT2BFiT participants

The TXT2BFiT study was conducted with healthy, non-pregnant 18–35 year-olds living in an urban environment in Australia recruited via primary care and print and electronic media [27]. Eligible participants had a BMI between 23.0 and 31.9 kg/m^2^ with dietary and physical activity behaviours that failed to meet national recommendations. Participants were required to have a mobile phone capable of receiving text messages and access to the internet at least weekly. Inclusion and exclusion criteria have been described in detail elsewhere [26]. Materials and methods of the TXT2BFiT 9-month trial were approved by the Human Research Ethics Committee in September 2012 (Approval number 15226).

### TXT2BFiT design

Details about the TXT2BFiT study protocol and recruitment are reported in separate publications [25–27]. In brief, the two-arm parallel RCT tested efficacy of our m-health lifestyle program for weight maintenance in young adults. The 9-month study consisted of a 3-month intervention period and 6-month maintenance period and it was conducted between November 2012 and April 2015. The 3-month TXT2BFiT intervention program comprised eight gender-specific weekly motivational text messages personalised according to stage-of-change for nutrition and physical activity strategies aimed to facilitate behaviour change around weight maintenance; weekly emails, five personalized coaching calls over 3-months; continuous access to purpose-designed smartphone applications; and community blog and support resources available on the study website. Two Accredited Practising Dietitians (APD) conducted the personalised coaching calls incorporating intervention participants’ stage-of-change and motivational interviewing techniques to set personal goals, discuss barriers and enablers, feedback on participant progress and set new goals. Participants set one goal to prevent weight gain and maintain their body weight within the normal BMI range (18.5–24.9 kg m^−2^) or to move towards a normal range. Participants also set between one and three goals relating to their diet or physical activity. All goals set over the five coaching calls and two booster calls were participant directed with dietitian input if required. Intervention participants were also mailed an 18-page printed booklet that included dietary information and sample meal plans as well as a two-page handout summarizing the Australian National Dietary and Physical Activity Guidelines given to controls [28, 29]. During maintenance phase, month four to nine, one monthly text message, one monthly email and two booster coaching calls at 5-months and 8-months were provided to intervention participants.

Control participants received the mailed two-page handout only, one introductory call (week 0) to introduce the program (no coaching given), four text messages i.e. one every three weeks and access to a website with only electronic versions of the two-page handout, consent form, study information statement and contact information.

All participants enrolled in the TXT2BFiT study were sent a link to online surveys at 3-months and at 9-months that included questions on use, frequency, timing, difficulties and overall experience with all program components. Control participants were asked similar questions for the minimal components of the program they were exposed to (text messaging and website use) for consistency of survey method.

### Frequency of use of components and frequency for number of components used

All participants completing the online surveys were asked “what parts of the program did you use?” This included use of the components i.e. coaching calls, text messages, emails, smartphone applications, printed booklet, community blog and each of the seven resources available on the website. Resources included “easy, healthy eating on a budget”, “emergency meal tool kit”, “meal planning worksheet”, “commit yourself: physical activity planner”, “tips for take-out meals”, “seasonal guide to fruit and vegetables” and “staying healthy over the holidays”.

### Dose delivered and engagement with program components

Data on dose delivered and engagement with program components were continuously collected for monitoring purposes for the duration of the study and stored in a database. Data included completed coaching calls, text messages delivered and replied to, emails delivered, download and logins to smartphone applications, and posts on the community blog.

Coaching calls were considered completed and engaged with, if all main aspects of the coaching call guide had been delivered to the participant (previous behavioural goals assessed and new behavioural goals set if appropriate). Delivery reports were downloaded from the text message web platform (MessageMedia®) and data synthesized on number of text messages delivered and on the number failed to be delivered per participant (dose delivered). Intervention participants were asked to reply ‘OK’ to 16 text messages during the intensive phase (months 1–3) and to each of the six text messages sent during the maintenance phase (months 4–9), while control participants were asked to reply ‘OK’ to all four text messages sent during the intensive phase. Engagement was determined by the number of replies per participant. Emails were considered delivered if no undelivered email was returned (dose delivered). Smartphone application downloads (dose delivered) and logins (engagement) were retrieved, as were details of the posts by researchers and participants on the community blog section of the website (engagement).

### Frequency, timing and difficulties experienced with program components

The delivery frequency of the text messages and emails were assessed by asking “were text messages/emails sent often enough?” with an option to report the number of text messages or emails per day they would have preferred to receive. Preference for the delivery time of text messages and emails were assessed by asking “was the time of day that text messages/emails were sent appropriate?” Participants were also asked to report any difficulty experienced using the text messages, emails, smartphone applications or website.

### Overall perceptions of program components

The online surveys asked participants if they had any other comments about the TXT2BFiT program. In addition, between March 2014 and April 2015 all individuals, who participated in the TXT2BFiT intervention arm were invited via email at the completion of the intervention to participate in the semi-structured telephone interview qualitative sub-study. The process evaluation sub study was approved by the Human Research Ethics Committee in February 2014 (Approval number 2014/091). The aim of the semi-structured interviews was to explore participant's experiences with and use of program components to corroborate the quantitative effects observed. Participants were asked questions about which aspects of the program they found useful in helping them achieved their goals and which aspects of program could be improved for future study implementation.

Both participants who completed or did not complete the 9-month TXT2BFiT intervention arm were contacted by email, but participants who had officially withdrawn were excluded. Participants responded via email and gave informed consent. Those who did not respond were followed up by a telephone call within two weeks of the email. If they did not respond to the telephone call, no further contact was made. The semi-structured interviews were administered by telephone, lasting approximately 15–20 min and participants were reimbursed for their time with a small AU$10 gift voucher. The interviews were audio recorded for the purpose of transcription and data analysis.

### Data sources, analysis and statistics

A summary of all process evaluation data and their definitions is provided in Table 1. Descriptive statistics were reported for quantitative data. De-identified qualitative data were coded using the NVivo Software program (QSR International Pty Ltd. Version 10, 2012, Victoria, Melbourne). One researcher (SP) trained in qualitative research methods conducted, transcribed and coded all participant comments and interviews, allowing for data immersion and obtaining an overall sense of the data. Content inductive analysis was used for each mHealth component, and categories ranked per mHealth component [30]. An open coding approach was adopted, forming a general description of the research topic through generating categories and subcategories as they emerged [31]. This systematic approach is appropriate for semi-structured interviews to determine trends and patterns. Discussion with a second researcher (MA-F) familiar with the data finalised and confirmed emerging categories. Verbatim quotes from interviews that best represented the key findings for each category were highlighted for subsequent reporting purposes.

**Table 1:** Summary of data sources and definitions

Quantitative data
Use of components and frequency for number of components used: • Post-intervention and follow-up survey data
Frequency, timing and difficulties experienced with program components: • Post-intervention and follow-up survey data
Dose delivered and engagement with program components: • Coaching calls completed (dose delivered and engagement = number of coaching calls completed) from researcher call log database • Text messages delivered (dose delivered = number of text messages delivered) and replied to (engagement = number of text messages replied to) from MessageMedia® web platform • Emails delivered (dose delivered = number of emails delivered) from researcher email log database • Smartphone application downloads (dose delivered = number of downloads per app) and logins (engagement = number of logins per app) from app developers database • Website community blog posts participants (engagement = number of participant posts on the community blog) from TXT2BFiT website
Qualitative data
Explore participant’s experiences with and use of all program components and their usefulness in helping them achieved their goals and future improvements: • Semi-structured telephone interviews conducted at end of the maintenance phase with intervention participants • Post-intervention and follow-up survey data

## Results

### Participants’ profile

The total randomized sample were aged 27.7 ± 4.9 (SD) years, female (61 %, 152/248), English speaking only (69 %, 172/248), university educated (62 %, 153/248) and living in a socio-economically advantaged area (75 %, 187/248). Participants were overweight on the basis of BMI classification (BMI 27.1 ± 2.5 (SD) kg m ^−2^). There was no significant difference in baseline characteristics between participants who completed post-intervention (3-months, intervention, *n* = 110, control *n* = 104) [26] and follow-up surveys (9-months, intervention, *n* = 96, control *n* = 104) and participants that did not.

### Frequency of use of components

**Fig. 1:**
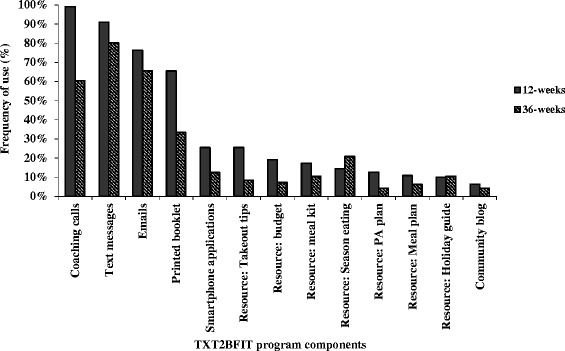
Use of all program components at post-intervention (3-months, *n* = 110) and follow-up (9-months, *n* = 96) in intervention group

Frequency of TXT2BFiT program component use for intervention participants at 3-months and 9-months is shown in Fig. 1. At 3-months coaching calls were used by all participants, deceasing to 60 % at 9-months. Text message and email frequency of use remained consistently high for the 9-month duration. The printed booklet was used by 66 % of participants during the 3-month intervention. Smartphone applications, resources and the community blog were used by less than 25 % of participants at both 3-months and 9-months.

At 3-months 35 % of control participants reported using the two page handout summarising the Australian National Nutrition and Physical Activity guidelines and 78 % reported using the four text messages (data not shown).

### Frequency for number of components used

**Fig. 2:**
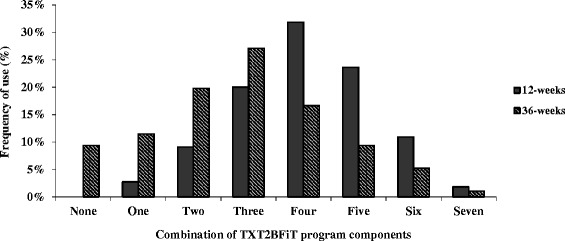
Frequency for number of components of the TXT2BFiT program assessed at post-intervention (3-months, *n* = 110) and follow-up (9-months, *n* = 96) in intervention group. (Website resources were treated as one component and participants using ≥ 1 were allocated one component)

At 3-months, 32 % of intervention participants reported using any combination of four program components, with 75 % or higher using three to five components (Fig. 2). This decreased to three components at 9-months (27 %), with more than 60 % using two to four components.

### Dose delivered and engagement with program components

#### Coaching calls

Overall 92 % (574/625) were completed at 3-months and 81 % (187/230) at 9-months (Table 2). Intervention participants completed on average 4.6 of five coaching calls, with 82 % completing all five at 3-months. Intervention participants completed 1.6 of two coaching calls on average, with 77 % completing both between 4-months and 9-months. All control participants completed the introductory telephone call.

**Table 2:** Participant engagement, dose delivered, frequency, timing and difficulties experienced of TXT2BFiT program components by intervention groups

Program component	Intervention		Control	
	Post intervention 3-months^a^	Follow-up 9-months^a^	Post intervention 3-months^a^	Follow-up 9-months^a^
Coaching calls				
Dose and engagement				
Completed, % (n)	91.8 (574/625)	81.3 (187/230)	100 (125/125)^b^	n/a
Text messages				
Dose				
Delivered, % (n)	97.8 (11543/11808)	95.7 (660/690)	97.0 (485/500)	n/a
Engagement				
Replying to ≥ 50 %, % (n)	52.8 (66/123)^c^	40.0 (46/115)^d^	91.2 (114/125)^e^	n/a
Replying to all, % (n)	20.3 (25/123)	6.1 (7/115)	62.4 (78/125)	n/a
Frequency (sent often enough) % (n)	95.5 (105/110)	75.0 (72/96)	n/a	n/a
Timing (suitable), % (n)	93.6 (103/110)	n/a	n/a	n/a
Difficulties, % (n)	8.2 (9/110)	4.2 (4/96)	1.9 (2/104)	n/a
Emails				
Dose				
Delivered, % (n)	100 (1476/1476)	100 (690/690)	n/a	n/a
Frequency (sent often enough), % (n)	92.7 (102/110)	71.9 (69/96)	n/a	n/a
Timing (suitable), % (n)	96.4 (106/110)	n/a	n/a	n/a
Difficulties, % (n)	3.6 (4/110)	3.1 (3/96)	n/a	n/a
Smartphone applications^f^				
Dose and engagement				
Downloads, % (n)				
ePASS	52.0 (64/123)		n/a	n/a
eSIYP	30.9 (38/123)		n/a	n/a
eVIP	43.1 (53/123)		n/a	n/a
Logins, n (median, range)				
ePASS^g^	327 (3, 1–39)		n/a	n/a
eSIYP^h^	128 (2, 1–28)		n/a	n/a
eVIP^i^	219 (2, 1–39)		n/a	n/a
Difficulties	28.2 (31/110)	19.8 (19/96)	n/a	n/a
Website				
Engagement				
Community blog posts by staff, n	10		n/a	n/a
Community blog posts by participants, n	8		n/a	n/a
Difficulties, % (n)	7.3 (8/110)	3.1 (3/96)	2.9 (3/104)	n/a

^a^110 intervention and 104 control participants completed the 3-month post-intervention survey and 96 intervention and 104 control participants completed the 9-month follow-up survey

^b^Introductory phone call only, no coaching given

^c^replying to ≥ 8 text messages

^d^replying to ≥ 3 text messages

^e^replying to ≥ 2 text messages

^f^Data for smartphone application eTIYP (take-out food app) was lost due to a change in server by external provider during the RCT

^g^ePASS, physical activity app

^h^eSIYP, sugar-sweetened drinks app

^i^eVIP, fruit and vegetable app

#### Text messages and emails

Text message delivery was 98 % (11543/11808) during the 3-month intervention period, and 96 % at 9-months (660/690) (Table 2). Text messages requiring a response during the first 3-months (16 text messages), were responded to an average 8.8 times, with 20 % of participants replying to all. All maintenance messages during months four to nine requested a response (six text messages), and reply declined to an average 1.9 times and 6 % replied to all. Email delivery was at 100 % for the duration of the study, with no email undelivered (Table 2).

Of the 500 control text messages scheduled 3 % were not delivered. Most control participants replied to two or more of the four text messages (114/125, 91 %), with 62 % (78/125) replying to all four text messages (Table 2).

#### Smartphone applications and website

Slightly over half (52 %) of participants downloaded the smartphone applications and less than half (43 %) of participants reported using the website (Table 2). The monitored community blog was the least used component, with only four participants contributing and ten posts made. Topics included shared experiences, tips and questions.

### Frequency, timing and difficulties experienced with program components

#### Text messages and emails

Participants reported text messages and emails were sent often enough during the 3-month program (96 and 93 % respectively) (Table 2). The vast majority were satisfied with the timing of the text messages and emails (94 and 96 % respectively). A very small number reported delays in delivery of text messages due to service provider error and delays in delivery of emails (going to junk folders as an example).

#### Smartphone applications and website

Difficulties reported with program components were highest in the smartphone applications (28 % of participants) (Table 2). Participants described the applications as “clunky” and hard to navigate. Difficulty in initially downloading the smartphone application was the next most reported problem and followed by difficulty logging in. The design and difficulty issues participants reported contributed to their low app usage. A small number reported difficulty with website login and accessing the healthy weight tracker.

### Overall perceptions of program components

Fifty intervention participants at 3-months and 40 intervention participants at 9-months completed the open ended survey question asking “did you have any other comments about the TXT1BFiT program?” (Data not shown). Feedback was positive overall, with the coaching calls referred to as the most helpful component due to the accountability and motivation elicited by the coaching calls. Suggestions included further personalisation of other components, such as the text messages and emails.

Over two-thirds of control participants provided comments in both the 3-month (*n* = 40) and 9-month survey (*n* = 38). Control participants reported more contact was required to increase motivation and the program as they received it was not helpful. The “lack of regular support” was reinforced with 9-months comments, however, some participants mentioned it was the “kick start” required for a healthier lifestyle.

#### Personal goal of participation in the program

Ethical approval was granted after 42 eligible intervention participants had completed the intervention and were outside the two-week window required for participation in the process evaluation interviews. Seventy-three intervention participants were invited to take part in semi-structured telephone interviews. Thirty participants (41 %, (30/73) participation rate) accepted the invitation (13 males, 17 females). Intervention participants who took part in the semi-structured telephone interviews did not differ from those who did not take part. Participant perceptions of the program are presented in Table 3.

**Table 3:** Components, categories and attributes from the semi-structured interviews with TXT2BFiT participants^a^

Component	Category	Attributes	Verbatim quote (Gender, Age)
Personal goal of participation in the program	1. Weight loss	Health, sustainable	“So I guess weight reduction as my main goal…. But having a healthy lifestyle also”. Female, 30
			“I have put on a few kilos… I want to lose the weight and lose it in a heathy way. “[I wanted] a program that is manageable in the long run, there are many diets that you can go on, but after a few months you give up. I didn’t want to go on a diet, but more like a long lasting change” Male, 33
	2. Improve physical activity behaviours	To lead to weight loss and/or weight maintenance, health, forming habits, sustainable, long-term	“Just to get into better habits, to maintain exercise habits [was] the main one”. Female, 27
	3. Improve eating habits	To lead to weight loss and/or weight maintenance, health, forming habits, sustainable, long-term	“I hoped to lose some weight and I guess generally get a better idea of healthier eating and good eating behaviours”. Female, 30
	4. Long term change	To lead to sustained weight loss and/or weight maintenance, behaviour maintenance, long term heath	“I need guidance to ensure that that the weight loss that incurred will stay off for a much longer time, so I needed help changing my daily habits” Male, 33
Most helpful	1. Coaching calls	Professional, accountability, motivation, personalised, non-judgemental	“I liked the phone calls and how they were individualised and I was talking to the same person each time, you get to know them, I found that helpful, being able to talk to someone on a regular basis who knew where I was at the last time and what was going on”. Female, 24
	2. Text messages	Practical tips and hints, easy to implement ideas, reminders, greater personalisation	“I guess the second part for me was the text messages, I thought they were good as well… they weren’t too much and they weren’t too little”. Male, 32
Least helpful	1. Smartphone applications	Logging in difficulties, navigation issues, design issues, functional capabilities	“I didn’t use the website, I tracked my weight in other ways [through My Fitness Pal] tracking my weight through the website wasn’t practical for me, mainly, because I was trying to access it through an iPhone, so it was fiddly, in fact a lot of the apps were useable, but they were fiddly”. Male, 29
	2. Website	Logging in difficulties, functional capabilities	
	3. Sudden decline in program contact	Program to maintenance phase change in contact	“… The gradually less contact, after the first 3 months, it became hard, some more continuing help”. Male, 24
Coaching calls	1. Professional	Appropriate qualifications, listened and understood concerns	“I thought the expertise of the nutritionist that was involved was very good, the advice was well planned out and well scoped. I had questions ready for them and I found that really useful because it was expert advice”. Male, 32
	2. Accountability	Someone checking in, reflection	“The calls were fantastic. It was cool to have someone check in and bounce ideas off, set goals and it was all really cool sensible stuff”. Male, 27
	3. Motivation	External motivation, support	“It was good because you want that accountability of an extra person aside from yourself at the beginning stages as a sounding board and external motivator”. Female, 27
	4. Personalised	Participant directed, collaboration	“I felt very positive and empowered, I guess because they relayed information I could really use and actually do something with. For example, when you look for healthy information you can be quite overwhelmed and don’t know where to start, whereas the coaching calls it was very focused…. I liked that [the calls] were very specific and focused on what I could do”. Female, 22
	5. Non-judgemental	Trust established, supportive environment	“… No question was too silly to be asked and discussed. I never felt like I couldn’t ask them anything”. Female, 27
Text messages	1. Tips and hints	Practical, non-fact based	“Some of the text messages that gave me ideas on how to actually incorporate increased fruit or increased vegetables or increased daily activities, specifically how to increase that in your daily life was really helpful, some of the messages that were intended to be motivational, such as the reasons to increase fruit and vegetables or increase my exercise were good in motivation, but I didn’t think they were as helpful, because we know it is good to be healthy, [I] just have trouble being healthy.” Female, 22
	2. Ease of implementation	Achievable suggestions, sensible	“Any that suggested a practical tip… something that I could implement straight away…. The ones that were helpful were the ones that gave you a specific task to do that day”. Female, 24
	3. Personalised	More personally relevant content, further targeting	“Some messages told me about things I was already doing, I have given that information in the baseline interview….I have no soft drink, that data was available and should have been personalised a bit more if possible”. Female, 32
	4. Reminders	Appropriate timing, habit forming	“I thought they were all sort of reminder ones…. They were sent at good times, like before I was about to buy something for dinner or eat my lunch”. Male, 32
	5. Small amounts of information	Easy to read, appropriate delivery medium, increased chance of reading content and/or adapting the targeted behaviour	“I really liked the [text messages], they were constant and I didn’t have to go out of my way to get them, they popped up and were helpful and gave good tips…” Female, 30
Emails	1. Limit	Limited to coaching call summary email	“When I got the more personal ones, like after the nutritionist coaching call, which contained the goals we discussed, I printed them out and put them on the wall at my office, so that worked quite well, but for the other ones… it might be check out the website, or we have some new tools, but I was just too busy to follow-up with that”. Female, 30
	2. Greater personalisation	Coaching call summary email increased change of reading content as personally relevant	“I think the emails were the aspect I dismissed the most easily, as I do get a lot of emails during the day”. Female, 33
	3. Larger amounts of information	Include links to program resources personally relevant to the participant	“[Emails] are flexible, as they give you heaps of different things to read, but it wasn’t enforced and I could work it around my own time, and that flexibility still made it good to get the help that I needed without the feeling that I had to go out of my way to do it”. Male, 28
Website and smartphone applications	1. Comparable to commercially available smartphone applications	Reverted to other commercially available smartphone applications	“I decided that I already have apps on my phone that I use to track diet and exercise, so I kept using those ones…. I think it was the usability of the TXT2BFiT ones, especially trying to use [the apps] on my phone, I found them a bit difficult to navigate” Female, 32
	2. Improved self-monitoring capabilities	Reverted to other commercially available self-monitoring devices	
	3. Easily accessible	Change to standalone non web-based applications, more user friendly mobile website, contain personal information from coaching calls (not available in other applications)	“…. They weren’t particularly intuitive or user friendly, but the content was useful once I got in, but the usability was the issue. I suppose I was looking for a central place where I could record all my information and where I could get more information, I was expecting the apps to be more user friendly and more practical.” Male, 29
External motivators	1. Commercially available smartphone apps	My Fitness Pal, Map My Run	“My Fitness Pal, I used this to set my goals with [the dietitians], it is very easy to use, you can scan food items and learn what you are eating, so you can easily keep an eye on how much you have eaten, but also see the proportions of the food you have eaten in relation to your daily goals”. Male, 33
	2. Commercially available self-monitoring devices	Fitbit, Jawbone	“I got a FitBit and that tracks steps and sleep and links to My Fitness Pal so that has been helpful to reach my goals”. Female, 32
	3. Social support	Family, partners, gym memberships, sporting groups	“Two friends of mine, we put a hard line of [getting] a six pack by Christmas, so that was a motivating factor, so going to the gym with them and working together definitely helps, so it was motivating for me to have that social support, it helped a lot”. Male, 33
Improvements	1. Greater personalisation	Text messages, emails, website	“It does need more tailoring…. More individual or specific, more personalised and more specific to the goals I was trying to achieve… it would give me more of sense of accountability”. Female, 24
	2. More centralised	Smartphone application consolidation (4 into 1), personal goals available from coaching calls to track progress	“I found having all of my data in one central location motivating, as I could see how much exercise affected how much I ate for the day and it also have a pedometer in it, so as long as I had my phone with me I could count how many steps I took that day and when I stood on the scales it would send it to my phone and I would have my weight and my body fat content there and everything was connected and in the one place and that was really helpful”. Female, 23

^a^30 intervention participants completed the semi-structured telephone interviews at 9-months

Weight loss was the primary reason for enrolling in the TXT2BFiT program, with some participants mentioning sustainable weight loss (Table 3). Improving physical activity and eating habits to improve health were commonly mentioned mostly in combination. Participants used words such as ‘habits’, ‘behaviours’, ‘sustainable’, ‘long-term changes’ and ‘maintain’ in relation to reducing weight, and improving physical activity and eating habits.

#### Coaching calls

The coaching calls were reported as the most helpful component (Table 3). Participants thought the calls were professional and personalised, acting as an external motivator. They described how the information was “all really cool, sensible stuff” and it was important for them to be consulting with a trained professional (APD). Accountability was a common theme. Participants mentioned a positive sense of accountability to the APD, and how someone “checking in” and “to bounce ideas off” helped them towards achieving their goal(s). Participants felt they could direct the coaching calls to meet their requirements, but they also mentioned how the calls were a collaboration and if an approach or goal wasn’t working for them, they could re-work their goal with the APD and it was within a non-judgmental environment.

#### Text messages

Participants liked the text messages as they provided “sound bite information” (Table 3). Tip and hint based text messages were preferred by participants as they were practical. Fact based messages were less preferred because most participants acknowledged that their nutrition and physical activity issues were not from a knowledge deficit. The easier the text messages were to implement into daily life, the more achievable and sensible they were perceived. The four behaviours targeted in the text messages were not relevant to all participants, and greater personalisation was suggested. However, despite some text messages being irrelevant to the participant’s goal, they still regarded them as a reminder. Participants were reminded of targeted behaviours at appropriate times (i.e. energy dense take-away consumption on a Friday and Saturday evening at 5 PM), which they described as contributing to positive habits forming. Text messages were viewed as easy to read, brief information that was not burdensome to participants.

#### Emails

Participants associated emails with work and spam (Table 3). Most weekly emails were viewed as impersonal and only scanned over, but the email sent as a summary of the individual coaching call was well received. This email was personalised with the agreed upon goals for the next coaching call and contained personally relevant links to study materials (website resources and/or smartphone applications). As this email was anticipated, it was reported as always read, regardless of length and would often be printed out for display.

#### Website and smartphone applications

The least helpful component mentioned was the smartphone applications for reasons such as logging in difficulties and navigation issues (Table 3). Participants did not engage with the smartphone applications, as they did not compare to other commercially available smartphone applications. They expected greater self-monitoring capabilities linked to the goals that they were working on with the APD in the coaching calls. Similarly, the website was not frequently used and issues were raised around access and difficulty logging in. Participants said the resources were easily accessible on the website. However, most participants prefer to use their smartphone to access the website and a new mobile version was requested.

#### External motivators and improvements

Participants reported using commonly available smartphone applications and self-monitoring devices such as My Fitness Pal and Fitbit (Table 3). Furthermore, most had some social support, including that of family, partners or memberships to gyms or sporting groups. Participants reported preferring an individualised program, with coaching calls the central feature and text messages, emails and website components tailored to their personal goals. A need for a more gradual decline in contact between the 3-month program and the maintenance phase was commonly mentioned.

## Discussion

To our knowledge this is the first study to elucidate young adults’ perceptions of and engagement with a mHealth program. Not surprisingly we discovered the desire for personalised intervention, evident by the high value placed on the behavioural coaching calls with a dietitian and text messages and summarising emails tailored to their personal nutrition and physical activity goals. Participants would prefer that the four apps for self-monitoring and website resources all be incorporated into one smartphone application that can be individualised by entry of their personal data. The findings of this study will be used to revise TXT2BFiT for future users.

Findings from this process evaluation suggested the phone coaching calls were most valued and other mHealth components acted to supplement the coaching calls. Coaching calls were added to the program and the frequency of text messaging increased in response to feedback from our first pilot study. There is already well established evidence supporting the effıcacy of telephone delivered interventions to promote physical activity and dietary change [32]. Participants had clear goals of what they hoped to achieve during the program, which aligned with the aims of the TXT2BFiT study. Personally tailored eHealth programs with health professional input and use of a structured program has been shown to enhance weight loss results [33, 34]. Studies have suggested that successful interventions for the prevention of weight gain instil skills in goal setting, planning and self-monitoring [35] which were a key component of the coaching calls. The seven mHealth components of the program contained 18 (data unpublished) behaviour change techniques (BCT), of the 26 recommended for positive behaviour change [36]. Over 75 % of participants reported using a combination of three to five components in the intervention period, and therefore would have been exposed to a large proportion of these BCTs known to elicit change.

Text messages and emails were the other main aspect of the program participants found helpful to achieve their goals. While actually not a mobile component, the nutrition booklet was also engaged with and used by participants. Interventions with text messages components have been shown to be beneficial in relation to weight loss [15]. Participants preferred behaviour-based messages as opposed to knowledge-based messages. Practical behaviour-based messages were reported to be implemented frequently when received, and even more likely when the message was related to a goal discussed in the coaching call. This is in line with current evidence suggesting self-monitoring, goal checking, prompting behavioural cues and goal reset are important for behavioural maintenance [37, 38]. Benefits of text messages and emails are that they can be tailored by the health professional to meet the needs of the participant and supplement the human interaction of the coaching call and enhance effectiveness [39]. A recent eHealth mini-online education lessons intervention for young adults used stage-of-change targeted emails (‘nudges’) to reinforce the fruit and vegetable, physical activity and stress management behaviours targeted in the lessons. The process evaluation revealed most adhering participants were moderately motivated by the lessons, however, no process evaluation data was presented on the email nudges [40]. For the TXT2BFiT study, the text messages addressed only the four key lifestyle interventions: sugary beverages, take-away foods, fruit and vegetables and physical activity. The database of goals set during the five coaching calls will be analysed along with other avenues such as crowd sourcing [41] with an on-line community of young adults, to inform future additional message development and facilitate further personalisation.

Barrier and facilitators discussed in the coaching calls and identified in other young adult populations such as budget constraints, peer pressure and time management [42, 43] can be incorporated in future message content. Participants asked for a more gradual decline in contact frequency between the intervention and maintenance phase. Whether longer duration of delivery of program components could enhance the changes already made and could result in maintenance beyond 9 months of this study is yet to be discerned.

Several recent studies used process evaluations to determine factors related to mHealth [24, 26, 44, 45] and eHealth [23, 46] implementation in young adults and other populations. The community blog in this study was not engaged with by participants, however, recent research has suggested many participants may passively engage with social networking i.e. read blog posts, but not respond or contribute [24]. If the community blog posts by research staff had continued, a greater dose of the intervention component, or more participants reporting use may have resulted. Morrison et al., [45] showed that goal-tracking smartphones applications have the potential to improve individuals’ engagement with their personal health goals when used as a supplement to existing web-based intervention components consisting of weekly feedback on personalised healthy eating and physical activity plans. The smartphone applications offered self-monitoring features [47], but one app had been developed for each behaviour in isolation in the belief participants might work on only one or two behaviours at a time. As it was clear weight loss was a primary goal for many, energy balance was important so linkage between diet and physical activity data was desired. This was the reason they reverted to commercially available smartphone applications. Researchers in the UK have developed an acceptable and feasible smartphone application incorporating goal setting, self-monitoring of diet and activity, and feedback via one weekly text message [48]. This integrated smartphone application approach alone was effective in pilot testing for weight loss in 50 women over a 6 month period. As researchers it is difficult to offer comparable quality and features as in commercial smartphone applications. Creating partnerships with commercial providers could provide participants an evidence based program, designed using up-to-date technology, however, ethical issues arise in relation to data ownership. It should also be noted that the most popular app in Australia that was reported as being used in this study failed to produce weight loss over a 6-month period when trialled in the primary care setting in the US [49]. Self-monitoring smartphone apps designed by researchers, however, have demonstrated weight-loss efficacy [50]. The qualitative research does indicate that participants prefer smartphones for delivery of their intervention, including the phone feature in particular, so that moving all resources from the website into an app format would meet with approval.

### Strengths and limitations

This study provides insights not yet reported in mHealth literature. Qualitative studies using process evaluation to inform recruitment strategies [51], and understand motivators and barriers to behaviour change [42, 52, 53] for weight management in young adults is emerging. However, qualitative process evaluation research has been lacking in weight gain prevention interventions for young adults. A strength of this process evaluation is its comprehensiveness. It incorporates of multiple sources of data, measuring a range of process evaluation dimensions and indicators such as dose delivered and engagement. It also measures the different program components, which is very important considering the multicomponent characteristics of the TXT2BFiT program. Retention was high in this study with more than 80 % of participants completing the online surveys at 9-months. Despite our efforts, we were unable to contact participants lost to follow-up (i.e. not completing survey but did not officially dropout) if it was due to the delivery of the mHealth program. Research from an older rural population suggests unrealistic program expectations may inhibit initiation and continuation of behaviour change for weight management [54]. For the qualitative interviews, 42 participants were not contacted for telephone interviews due to delay in ethical approval. Lastly, the population in this intervention were mostly well-educated and from higher socio-economic backgrounds. Young adults less educated and from lower socio-economic backgrounds may require education-based health messages to improve nutrition knowledge.

## Conclusions

Obesity is a multifaceted public health issue; young adults are at increased risk, and prevention requires personalised solutions. mHealth programs have the benefit of offering the participating individual multiple components, thus being able to cater for a wide-range of needs. The TXT2BFiT has demonstrated efficacy in reducing and/or maintaining weight and improved dietary and physical activity behaviours. Our process evaluation has allowed a comprehensive understanding of use and preference for different program components. Future work will be required to translate the array of TXT2bFiT components to meet the needs of most young adults, to support them to stay healthy and active.

## Abbreviations

APD: Accredited practising dietitian
BCT: Behaviour change techniques
eHealth: Electronic health
mHealth: mobile health
RCT: Randomised controlled trial
USA: United States of America
